# The role of Th/Treg immune cells in osteoarthritis

**DOI:** 10.3389/fimmu.2024.1393418

**Published:** 2024-09-19

**Authors:** Zhi Wen, Liguo Qiu, Zifeng Ye, Xuyi Tan, Xiaotong Xu, Min Lu, Gaoyan Kuang

**Affiliations:** ^1^ Department of Joint Orthopedics, The First Hospital of Hunan University of Chinese Medicine, Changsha, Hunan, China; ^2^ Graduate School of Hunan University of Traditional Chinese Medicine, Changsha, Hunan, China; ^3^ Department of Joint Orthopedics, The Affiliated Hospital, Hunan Academy of Traditional Chinese Medicine, Changsha, Hunan, China

**Keywords:** OA, T cells, Th1/Th2, Th17/Treg, immune cells, inflammation

## Abstract

Osteoarthritis (OA) is a prevalent clinical condition affecting the entire joint, characterized by its multifactorial etiology and complex pathophysiology. The onset of OA is linked to inflammatory mediators produced by the synovium, cartilage, and subchondral bone, all of which are closely tied to cartilage degradation. Consequently, OA may also be viewed as a systemic inflammatory disorder. Emerging studies have underscored the significance of T cells in the development of OA. Notably, imbalances in Th1/Th2 and Th17/Treg immune cells may play a crucial role in the pathogenesis of OA. This review aims to compile recent advancements in understanding the role of T cells and their Th/Treg subsets in OA, examines the immune alterations and contributions of Th/Treg cells to OA progression, and proposes novel directions for future research, including potential therapeutic strategies for OA.

## 1 Introduction

Osteoarthritis (OA) is a common joint disease characterized by the degeneration of the articular cartilage. OA mainly involves the knee joint, hip joint, and distal interphalangeal joint. The articular cartilage, subchondral bone, ligament, joint capsule, synovium, and muscles around the joint are typically affected (1–3). Globally, OA is recognized as one of the main causes of morbidity and disability (4, 5). It has been estimated that by 2032, the proportion of people, aged 45 years and over, medically diagnosed with OA will increase from 26.6% to 29.5% (knee osteoarthritis (KOA) from 13.8% to 15.7%, and hip osteoarthritis from 5.8% to 6.9%) (6). In the early stage, OA is characterized by increased bone remodeling, a loss of bone structure, and slow subchondral bone densification (7). Chronic intra-articular inflammation and cartilage degeneration follow. Eventually, intractable joint pain and joint deformities occur, which in turn, seriously affect the quality of life and the ability to work of the patients (8, 9). Traditionally, OA was considered a non-inflammatory disease involving anatomical joint damage and reduced function caused by cartilage degeneration. The pathogenesis was mainly attributed to age, body mass, sex, and abnormal joint loading, as well as articular cartilage damage caused by joint injury, misalignment, and other mechanical factors (10, 11). Most scholars have focused on the molecular biology of promoting cartilage interstitial synthesis, inhibiting cartilage stroma decomposition, and inhibiting chondrocyte apoptosis, as well as the biomechanics of joint injury and tissue engineering for promoting cartilage repair. To date, the potential mechanism of cartilage repair remains unclear (12–14). While researchers have acknowledged the significance of cartilage degeneration in the development of OA, there is still limited understanding of the concurrent inflammatory reaction. More in-depth research on the pathogenesis of OA is needed to advance clinical treatment. More recently, the pathophysiology of OA has shifted from a degenerative “wear” disease of the articular cartilage to being recognized as a multi-factorial disease involving all joint tissues, with an underlying complex pathophysiology (15). Although OA has historically been defined as a type of non-inflammatory arthritis, many patients with OA exhibit inflammation-related symptoms, such as morning stiffness, fever, pain, and joint effusion. Increasing numbers of studies have shown that the inflammatory mediators produced by the synovium, cartilage, and subchondral bone are associated with cartilage injury in the pathogenesis of OA (16). Therefore, OA is becoming more recognized as a systemic inflammatory disease. Moreover, OA is being described as a persistent state of low-grade inflammation, rather than a passive degenerative disease or so-called abrasive disease (17, 18).

A variety of immune cell infiltration is found in the synovium of patients with OA. This established that a relationship exists between orthopedic presentations and immunology. Consequently, the pathogenesis of OA involves immune inflammatory reactions, and thus, can be classified as bone immunology dysfunction (19, 20). The continuous intersection between immune cells and bone metabolism has attracted more and more attention to bone immunology. An understanding of the relationship between immune cells and bone metabolism is warranted. In patients with OA, the synovium often shows inflammatory cell infiltration. At present, T cell, B cell, macrophage, mast cell, and NK cell infiltration have been most commonly found in the synovium of patients with OA (21, 22). Moreover, innate immune components, such as complements, macrophages, proinflammatory cytokines, and chemokines, as well as adaptive immune cells, such as T cells and B cells, play important roles in the development of OA (15, 23, 24). Although the specific pathological mechanism of T cells in OA is not clear, the OA synovium has been shown to possess a greater abundance of T cells than a healthy synovium (20). OA has been associated with many types of T cells, including helper T cells (Th) and regulatory T cells (Treg), suggesting that abnormal Th/Treg cells may be an important factor in its pathogenesis (15). Hence, scientists are now gaining a better understanding and acknowledging the significance of immune cells like T cells in osteoarthritis. While some research has been conducted in the past, further in-depth studies on the mechanisms and pathophysiology are required to fully grasp their role. This review explored the progress of T cells and their subsets (Th/Treg) in OA, discussed the role and changes in T cells including Th/Treg in disease progression, and proposed future research directions and the potential for new OA treatment.

## 2 T cells and OA

**Figure 1:**
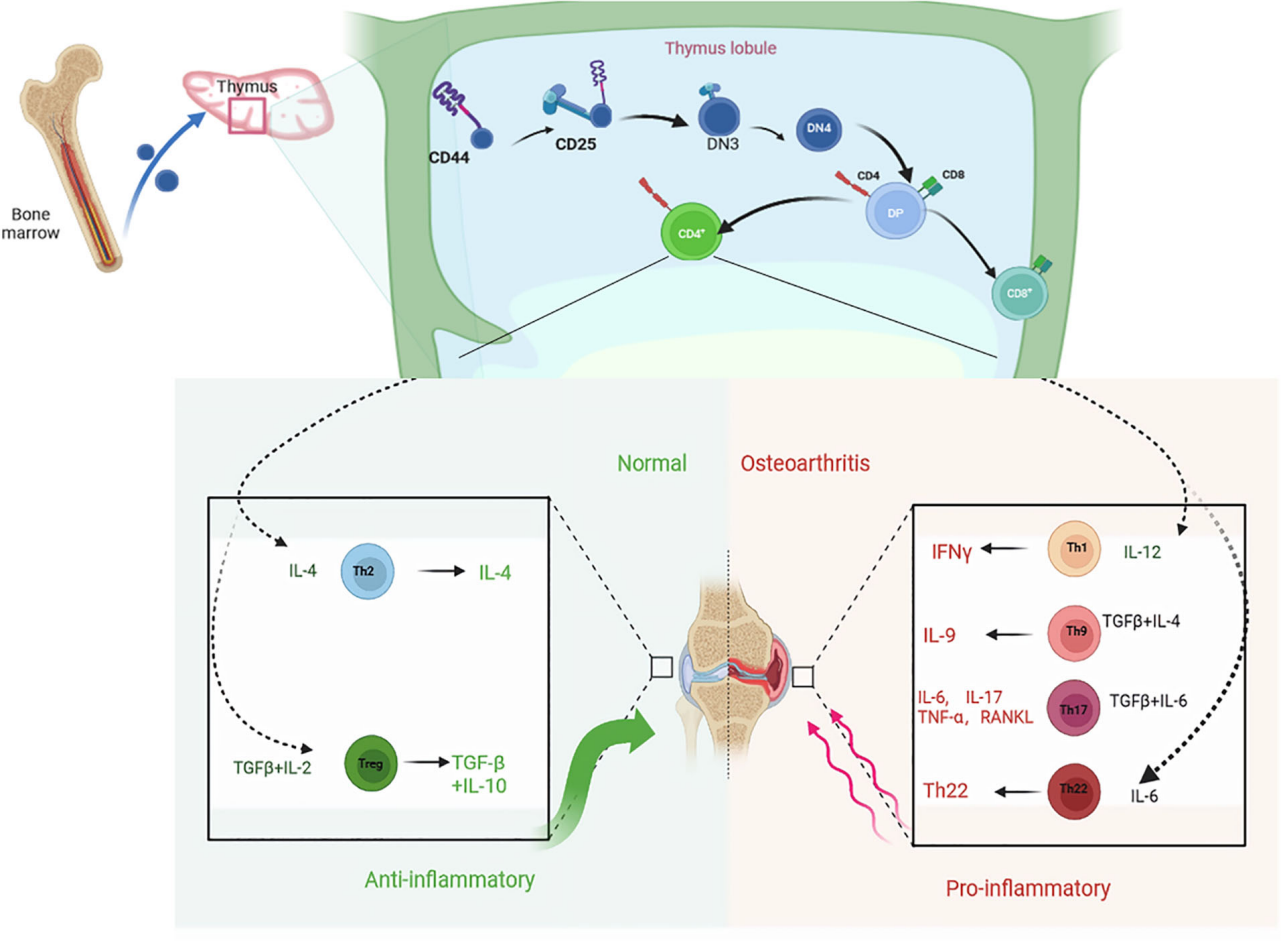
T cells originate from hematopoietic stem cells in the bone marrow and undergo differentiation, development, and maturation in the thymus. Differentiated into distinct cell subpopulations under the influence of specific thymus factors such as cytokines and hormones. Th2 and Treg cell subsets play an anti-inflammatory role by secreting cytokines IFN-γ, IL-4, IL-10 and TGF-β, delaying joint degeneration and cartilage injury, while Th1, Th9, Th17 and Th22 cell subsets promote joint inflammation.

T cells play an important role in maintaining body health and preventing diseases. They are the main cellular components of the adaptive immune system and are responsible for mediating cell-based immune responses to prevent the occurrence of various diseases (25). T cells originate from hematopoietic stem cells in the bone marrow and undergo differentiation, development, and maturation in the thymus, facilitated by the influence of specific thymic factors such as cytokines and hormones (**Figure 1**). T cells are effector cells involved in the adaptive immune response of proliferation, cytokine production, cytotoxicity, and differentiation (26). Under normal circumstances, the number of T cells and their subsets in the surrounding tissue is relatively stable. Immune abnormality is regarded as a change in the ratio or absolute value of the total number of T cells or their subsets. This immune abnormality is closely related to the occurrence and development of some diseases (27). It has been confirmed that pro-inflammatory cytokines play an important role in the pathogenesis of OA. Inflammatory responses aggravate the severity of OA by inducing cartilage degradation (28). The cell types involved in OA include osteoblasts, osteoclasts, chondrocytes, synovial fibroblasts, T cells, macrophages, and mesenchymal stem cells (MSCs) (29). Acquired immune cells, such as T cells, B cells, and NK cells, play an important role in the pathogenesis of OA (30, 31). In particular, T cells is critical in adaptive immunity. In the disease microenvironment, T cells are activated to produce a large number of cytokines and inflammatory mediators. Activated T lymphocytes are associated with the occurrence, development, and prognosis of OA (32, 33). Importantly, the immune responses associated with activated T cells or abnormal T cells are related to bone loss and bone destruction in arthritis. An increase in CD4+T, Th1, Th1/Th2 ratio, and Th17 enhance osteoclastic activities, while an increase in CD8+T cells, Treg, and CTLA-4 inhibit osteoclasts (34).Bone is a dynamic organ. It is in a dynamic equilibrium of continuous reconstruction or remodeling during the lifespan. To maintain the balance required in bone structure, osteocytes, osteoblasts, and osteoclasts coordinate and cooperate during bone remodeling (35). In arthritis, activated T cells regulate bone loss and joint destruction by regulating the equilibrium between the receptor activator of nuclear factor kappa-B ligand (RANKL) and osteoprotegerin (OPG). The expression of OPG in T cells is recruited by antigen receptors, indicating that activated T cells can affect bone metabolism through OPG and RANKL (36, 37). In a T cell-dependent mouse arthritis model, the blocking of RANKL with OPG can prevent the destruction of bone and cartilage, but cannot inhibit inflammation (38). Abnormal T cell immunity promotes the abnormal expression of inflammatory cytokines, such as TNF- α, which leads to osteoclast-mediated bone erosion and osteoporosis in autoimmune arthritis. Hence, it has become clear that the immune responses from activated or abnormal T cells induce bone loss and bone destruction in arthritis (39).

In the early stage of OA, inflammation occurs with inflammatory factor infiltration in the synovium. The degree of synovitis is closely related to the symptoms of OA and the progression of the disease (40, 41). Prior research has shown that the primary immune cells found in the synovial tissue, synovial fluid, and subpatellar fat pad of individuals with osteoarthritis are T cells, macrophages, and synovial tissue resident macrophages (STRM) (42–44). In the early stage of OA, CD4+T cells induce synovitis by secreting TNF- α and IL-6, and the levels of these cytokines are significantly correlated with pain and dysfunction clinically (45). In a study using mice with gene knockout of CD8+T lymphocytes and anterior cruciate ligament transection, Hsieh et al. (46) found that the proliferation, hypertrophy, and granulation of the synovial tissue decreased on the 90th day, suggesting that there is a correlation between T lymphocytes in the synovial tissue and the progression of OA, although the specific mechanism is not clear. Scholars have found that T cell recruitment may be related to the enzymatic process. Using enzyme-linked immunosorbent assay (ELISA) to detect the supernatant of synovial cells with T cell deletion, researchers have found that T cell deletion decreases matrix metalloproteinase (MMP)-1, MMP-3, and MMP-9 levels, indicating that activated T cells in the synovium can induce the release of MMP and accelerate the process of cartilage destruction (45). These results confirm that T cells can induce OA directly or indirectly by secreting cytokines. Further study into the relationship between T cells and OA may provide new ideas for enhancing the diagnosis and treatment of OA.

## 3 Th cells and OA

### 3.1 Th1 cells

Th1 cells are a lineage of the CD4 effector T cells, which can phagocytize and clear antigens by activating macrophages and other immune cells. Th1 cells play an important role in identifying and clearing intracellular pathogens, such as viruses and bacteria (47). Th1 cells mainly secrete IFN-γ, TNF-α, and IL-2 cytokines, which can promote the further proliferation of other Th cells, leading to cellular immunity (48, 49). Initial CD4+T cells differentiate into Th1 cells under the action of IL-12 and IFN-γ. Importantly, Th1 cells play an anti-intracellular pathogen role in infections (50, 51). IL-2 and TNF-α, secreted by Th1 cells, can activate osteoclasts (52). Moreover, TNF-α can delay osteoclast apoptosis, aggravate subchondral bone destruction, localize bone remodeling after bone destruction, and subsequently, lead to the formation of new osteophytes (53).In early OA, inflammation occurs in the synovium. Rosshirt et al. (33) analyzed synovium samples from 40 patients with early OA by flow cytometry. The results showed that chemokine receptor (CXCR3/CCR5), cytokine (interferon-γ, preferentially expressed in Th1 cells), and CD161 (preferentially expressed in IL-17-producing Th17 cells) were significantly increased, indicating that the infiltration of inflammatory Th1 cells in early OA. This direct cellular interaction, combined with humoral immunity, is involved in the pathogenesis of early OA. Timo et al (54) evaluated the pain and function of the knee joint in 47 patients with OA, who underwent knee arthroplasty. The patients’ peripheral blood (PB), synovium (SM), and synovial fluid (SF) were sampled and different Th subsets were analyzed by flow cytometry. The results showed that synovial infiltration of Th subtypes (Th1, Th2, Th17) was significantly related to OA-induced dysfunction. Additionally, infiltration of CCR5+ and CCR3+ Th cells in the synovium was associated with osteoarthritic knee pain and dysfunction. Lathrati et al. (15) detected Th1 cells in the peripheral blood of patients with hyaluronic acid injection by flow cytometry. It was confirmed that the level of activated Th1 cells in the treatment group was significantly higher than that in the healthy controls. Monasterio (55) confirmed that Th1 cells are enriched in OA lesions and that these cells may activate subchondral osteoclasts through the RANKL/RANK signaling pathway to accelerate the inflammatory response. Thus, resulting in further aggravation of OA in patients. In their ACLT model rat study, Castrogiovanni (56) intervened by using physical exercise as a treatment. Results from the synovial analysis revealed that the levels of IL-4 and IL-10 in the ACLT model rats were significantly higher than those in OA model rats, while the levels of TNF- α and MMP-13 were decreased. These studies confirmed that Th1 cells are closely associated with the entire OA process, especially in the early stage of OA. The presence of Th1 cells can accelerate joint inflammatory responses, leading to cartilage matrix degradation and destroying joint homeostasis.

### 3.2 Th2 cells

CD4+T cells differentiate into Th2 cells under the action of cytokines, such as IL-4. Activated Th2 cells can produce cytokines including IL-4, IL-5, IL-10, and IL-13. These cytokines can promote the proliferation of Th2 cells and inhibit the proliferation of Th1 cells (41, 57). However, the researchers found that only low levels of IL-4 and IL-10 could be detected in the peripheral blood and synovial fluid of patients with OA by flow cytometry (58, 59). In a study of 18 patients with OA and 13 patients with RA, the researchers found that IL-10 transcription could be detected in the synovium of patients with OA and RA, but IL-4 and IL-5 were not detected (60). In a study that examined chemokine receptors and T cells in OA, the researchers found that compared with paired bone marrow, the cells expressing CC chemokine receptor 2 (CCR2) in the peripheral blood were significantly up-regulated and T cells CXC chemokine receptor 3 (CXCR3) were significantly down-regulated. In contrast, CCR4 was not significantly up-regulated. These observations suggest a tilt in the Th2 phenotype of patients with OA (61). Another study (62) confirmed that calcitriol can affect the differentiation of T cell subsets by inhibiting the proliferation of immature CD4+T cells to Th1 cells and promoting the maturation of Th2 cells, thus affecting the balance between osteoblasts and osteoclasts. In addition, vitamin D3 increases the production of anti-inflammatory cytokines (IL-4, IL-5, and IL-10) by Th2 cells, while inhibiting their production of pro-inflammatory cytokines (IL-2 and INF- γ). In this way, vitamin D3 regulates the immune balance of Th1/Th2 and limits the destructive effect of Th1 cells on tissue. In a clinical study (63), Javad compared the peripheral blood of 40 patients with OA treated with the natural drug, Krocina TM (containing crocin) and compared the results with the same number of patients who took a placebo. Real-time quantitative polymerase chain reaction (RT-PCR) was used to detect the expression of T-BET, GATA3, ROR- γ t, and FOXP3 as transcription factors specific to T cell subsets. The results demonstrated that after Krocina TM treatment, the expression of related genes (GATA-3 and FOXP3) increased. The result was significant for GATA-3 but not FOXP3, indicating that GATA-3 is a unique transcription factor that can differentiate T cells into Th2 subsets. Furthermore, expression of the GATA-3 gene is significantly increased in patients with osteoarthritis after crocin treatment, suggesting that crocin can affect Th2 subsets and enhance the anti-inflammatory state. Although previous studies have shown that the levels of cytokines related to Th2 cells in the synovium, synovial fluid, and peripheral blood of patients with OA are low, while the expression of IL-10 is occasionally increased, these findings did not suggest that the inflammatory response of Th2 cells is not associated with the pathogenesis of OA (64). Th1 cells produce pro-inflammatory factors, such as IL-2, IFN-γ, and TNF- α, while Th2 cells produce anti-inflammatory factors, such as IL-4 and IL-10. Th2 cells promote tissue repair by secreting IL-4 to promote the function of M2 macrophages and inhibit cell-mediated production of Th1 cells. Hence, the responses of Th1 cells and Th2 cells are considered pro-inflammatory and anti-inflammatory, respectively (65). In individuals who are in good health, there is a delicate equilibrium between Th1 and Th2 cells that helps the immune system eliminate pathogens efficiently without causing too much inflammation. However, in cases of OA, this balance is frequently disrupted, leading to an increased Th1 response and a decreased Th2 response. In sum, an imbalance in the Th1/Th2 ratio can activate osteoclasts and accelerate the inflammatory response, resulting in cartilage matrix degradation and destroying the homeostasis in cartilage (66).

### 3.3 Th9 cells

Th9 cells are a subgroup of effector CD4+T lymphocytes, which are differentiated from initial CD4+T cells induced by cytokines, such as IL-4 and TGF-β, and can also be induced by TGF-β alone. Activated Th9 cells are mainly characterized by the production of cytokines, such as IL-9 and IL-10 (67, 68). Th9 cells mainly accumulate in the synovial fluid and peripheral blood of patients with OA. IL-9 can maintain and increase the pro-inflammatory environment of OA, which leads to the migration and proliferation of inflammatory cells (26, 62). A study of psoriatic arthritis (PsA) and rheumatoid arthritis (RA), with OA as the control group, found that IL-9 promoted the growth and survival of locally activated T cells in an inflammatory environment. Although there was far less IL-9 in OA synovium than in PsA and RA, some infiltration was observed (69, 70). The results found that the number of Th9 cells and the level of serum IL-9 in patients with OA were significantly higher than those in healthy individuals (71). IL-9 is also an important growth factor for T cells, mast cells, and hematopoietic stem cells, and can inhibit apoptosis. Kundu-Raychaudhuri et al (69) used Western blots to detect the signal proteins related to the survival of Th9 in the synovial fluid and peripheral blood of patients with OA. The results demonstrated a high level of IL-9 in the synovial fluid and peripheral blood and suggested that the high level of IL-9 was produced by the activation of purified CD3+T cells. It has been suggested that part or all of the IL-9 in the synovial fluid and peripheral blood of patients with OA comes from CD3+T cells. Qi et al. (72) detected the number of T cells in the peripheral blood of 25 patients with OA and 13 healthy controls by flow cytometry. The results showed that the number of Th9 cells in the peripheral blood of patients with OA was significantly higher than that of the healthy controls. The level of serum IL-9 was also higher than that of the healthy controls. Moreover, the number and level of Th9 cells were positively correlated with the osteoarthritis index score (WOMAC) of Ontario and McMaster University in patients with OA and also with their clinical symptoms and joint function. Hence, the number or level of Th9 cells has been suggested as a possible marker for judging the severity of OA. Furthermore, current studies have confirmed that Th9 cells show obvious activation and aggregation in the synovial fluid and peripheral blood of patients with OA (70, 73). Overall, IL-9 cytokines can stimulate inflammatory and autoimmune responses, promote chondrocyte apoptosis, and inhibit cartilage repair, thus aggravating OA. Research findings indicate that Th9 cells may play a significant role in the development of osteoarthritis (OA), providing insights into the impact of immune response and inflammation on OA. This suggests a potential novel treatment strategy involving the modulation of Th9 cell function to control inflammation and enhance the well-being of individuals with OA. Therefore, targeted therapy for Th9 cells may offer a potentially new treatment direction for OA.

### 3.4 Th17 cells

Th17 cells are a unique and important subgroup of Th cells. Their function depends on the ability of the immune system to produce and secrete key cytokines, such as IL-17, IL-21, and IL-22 (74, 75). Th17 cells differentiate from resting T cells in the microenvironment where TGF-β and IL-6 inflammatory factors coexist, and play an important role in immune responses, especially those associated with inflammatory injury relating to anti-extracellular pathogen infections, and mediating autoimmunity (76). Th17 cells also play an important role in OA. In patients with OA, the number of Th17 cells and the level of serum IL-17 are significantly higher than those in healthy controls (72). IL-17, a key factor produced by Th17 cells, can destroy homeostasis within the extracellular matrix. Notably, IL-17 is a key mediator in the pathogenesis of chronic inflammatory diseases and one of the central cytokines of arthritis. IL-17 contributes to joint inflammation by promoting the production of inflammatory cytokines and attracting additional immune cells, such as neutrophils (77). IL-17 induces inflammatory cytokines, including TNF-α, IL-1β, IL-6, and matrix metalloproteinases, that can aggravate joint destruction. IL-17 can also increase the expression of RANKL, thereby activating osteoclasts, resulting in joint bone loss (55, 78). The increased expression of Th17 cells was found in the peripheral blood of patients with OA, and the concentration of IL-17 in the serum and knee joint synovial fluid of patients with KOA was positively correlated with KOA severity (KL grade). The level of Th17 cells and their cytokines have been suggested as a potentially important index for evaluating the severity of OA (79, 80). The most direct impact of IL-17 is in cellular immune responses, along with the membrane surface antigens of chondrocytes and synovial fibroblasts. Together, they promote the infiltration and tissue destruction of many kinds of immune cells, participate in the proliferation, maturation, and chemotaxis of neutrophils, and co-stimulate the activation of T cells (81). Won et al. (82) collected peripheral blood mononuclear cells (PBMC) and SF (SFMC) from healthy individuals and patients with ankylosing spondylitis (AS). In this study, the effect of C chemokine ligand 20 (CCL20) on the migration of Th17 cells was verified by a cross-hole migration experiment. The *in vivo* effect of CCL20 inhibition was evaluated using a SKG mouse model, which is primarily a model for rheumatoid arthritis (RA), rather than OA. It was found that CCL20 could significantly reduce joint inflammation by affecting the migration of Th17 cells and inhibiting CCL20. Jung et al. (83) used the collagen-induced arthritis (CIA) model, which is a prototype RA model, rather than an OA model, to study arthritis in a mouse model by collagen. The proportion of Th17 cells in the spleen of normal and high salt diet mice was detected by flow cytometry, and the expression of IL-17 in the joint and intestinal tissues was detected by immunohistochemistry. The effect of sodium chloride on the differentiation of peripheral blood mononuclear cells into Th17 in CIA mice and the contents of sodium and IL-17 in the synovial fluid of these mice were analyzed. It was found that sodium chloride aggravated arthritis by promoting the differentiation of mouse Th17 cells in a dose-dependent manner.

Research has demonstrated that pharmacological agents, including steroids and anti-tumor necrosis factor inhibitors, can impede the differentiation of Th17 cells, consequently mitigating the symptoms associated with osteoarthritis (OA). Additionally, other biologic therapies, such as anti-IL-17 and anti-IL-23 antibodies, may also be relevant in the treatment of OA. While the majority of investigations have concentrated on the role of Th17 cells in rheumatoid arthritis (RA), current evidence indicates that targeting Th17 cells may represent a promising avenue for future therapeutic strategies in the management of OA.

### 3.5 Th22 cells

Th22 cells are a subgroup of cells differentiated from helper T cells under the action of IL-6, IL-1β, and TNF-α (84). Th22 cells mainly express cytokines, such as IL-22 and IL-13. These cells were named Th22 because of their ability to produce IL-22 is significantly higher than that of other Th subsets (85, 86). Many studies have confirmed the role of Th22 cells in immune and neoplastic diseases (87, 88). In one study, Th22 cells isolated from RA peripheral blood and monocytes were co-cultured with macrophage colony stimulating factor and nuclear factor receptor activator KB ligand. The results showed that Th22 cells were more effective in inducing osteoclast formation than Th1 cells and Th17 cells (85). Interestingly, Miyazaki et al. (89) observed significant infiltration of Th22 cells in the synovium of patients with active RA, but no similar phenomenon was found in patients with OA. In another study, the researchers found that the number of Th22 cells and the level of IL-22 in the peripheral blood of patients with RA and AS were higher than those of patients with OA and healthy controls. Lejon (90) analyzed the level of T cell subsets, the related cytokines, and clinical characteristics of patients with AS versus controls from northern Sweden, and confirmed that an increased Th22 level was related to AS. Ahmad et al. (91) used CXCR3-specific antagonist NBI-74330 to block T cell-mediated signal transduction in DBA/1J mice with collagen-induced arthritis. It was found that NBI-74330 could significantly reduce the expression of IL-22 mRNA in the knee joint tissue of CIA mice. The anti-inflammatory effect of NBI-74330 may be related to a reduction in Th22 cell expression. While direct evidence linking Th22 cells to OA is currently sparse, their established roles in synovitis and bone destruction in RA suggest that similar pathways may be at play in OA. However, further research is essential to explore and confirm any such associations between Th22 cells and OA. Th22 cells are known to promote inflammation and autoimmune responses by secreting cytokines like IL-22, which may have implications for the progression of OA in settings yet to be fully understood.

## 4 Th1/Th2 cells imbalance and its relation with OA

Th1 and Th2 cells are two types of CD4+ T helper cells that play distinct roles in the host immune response. Th1 cells primarily release IFN-γ and tumor TNF-α, which are known for their role in enhancing cell-mediated immune response and tissue inflammation. In contrast, Th2 cells predominantly generate IL-4, IL-5, and IL-13, which are responsible for regulating humoral immune response and the anti-inflammatory process (92). The balance between Th1 and Th2 cells is typically carefully controlled by the immune system to ensure its normal function. However, this balance is disrupted in various conditions such as rheumatoid arthritis (93, 94), asthma (95, 96), inflammatory skin disorders (97), and allergies (98). While there is no direct evidence linking Th1/Th2 cell imbalance to OA, this connection can be inferred by measuring Th1 and Th2 levels in OA patients. Imbalances in Th1/Th2 cells have been implicated in the pathogenesis of osteoarthritis, contributing to inflammation and disease progression (99). This suggests an increased propensity toward inflammation. Furthermore, researchers have observed a significant increase in the levels of Th1 cells and the pro-inflammatory cytokines they produce, such as IFN-γ and TNF-α, in the synovial fluid and synovium of OA patients (100). Teng (101) conducted an extensive examination utilizing bi-directional Mendelian randomization and Bayesian co-localization techniques. Their studies revealed that the upregulation of TNF-α, which is secreted by Th1 cells, stimulated the generation of various pro-inflammatory cytokines and inflammatory mediators. This process initiated a series of inflammatory reactions, leading to joint inflammation and cartilage degradation, ultimately contributing to the progression of osteoarthritis. The cytokines produced by Th1 cells have the ability to suppress the activation of Th2 cells and their associated anti-inflammatory responses (102). Subsequent research has shown a notable reduction in both the quantity and activity of Th2 cells in individuals with OA, leading to a diminished anti-inflammatory capacity and an increased prevalence of pro-inflammatory reactions, These shift in immune response dynamics hinders effective management of joint inflammation and tissue damage in OA patients (29). In chronic bone immune disorders, such as fatty-degenerative osteonecrosis (FDOJ), over 80% of a study’s 197 patients exhibited metastasis of Th2 cells. Among these, 167 subjects had an elevated Th1/Th2 ratio, suggesting that the dysregulation of Th1/Th2 cells plays a significant role in immune impairment (103).

**Figure 2:**
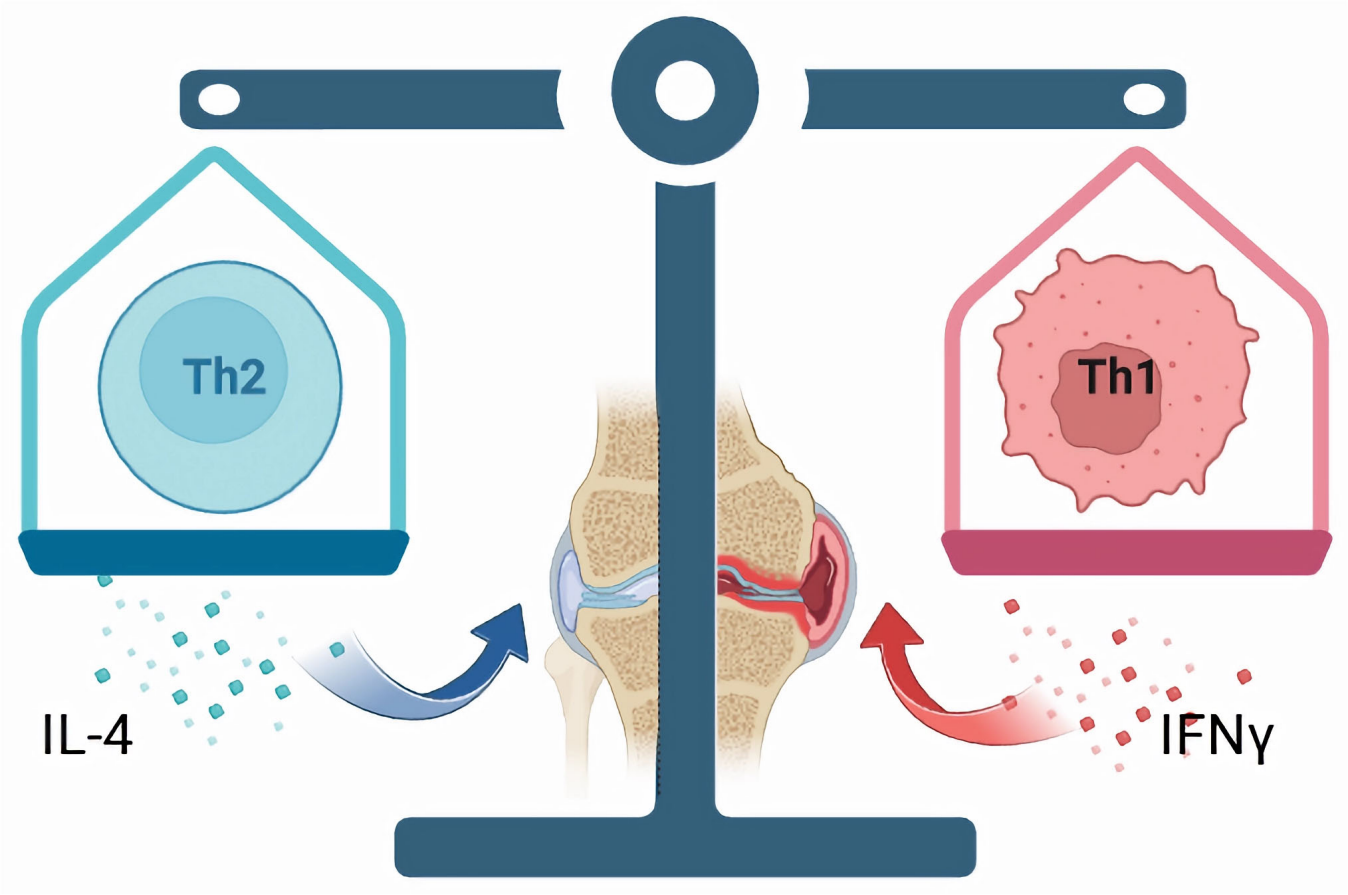
In individuals with OA, there could be a disparity in the ratio of Th1/Th2 cells, which is associated with the onset and progression of the condition. Th1 cells predominantly release IFN-γ, contributing to cell-mediated immune reactions and tissue inflammation, thereby facilitating the advancement of OA. Conversely, Th2 cells primarily generate interleukin-4 (IL-4), which plays a role in modulating humoral immune responses and anti-inflammatory mechanisms, consequently alleviating symptoms associated with OA.

The aforementioned research indicates a potential dysregulation of Th1/Th2 cells in both the synovial fluid and peripheral blood of individuals with OA. This imbalance is associated with the onset and progression of the condition. Given the significance of Th1/Th2 cell imbalance in OA (**Figure 2**), investigating the pathophysiological mechanisms of immune cells during the early stages of OA is highly valuable, as this phase presents the greatest potential for effective treatment and intervention. Rosshirt et al (33) conducted a quantitative analysis of the migration and activation of CD4+ T cells in peripheral blood (PB), synovial fluid (SF), and synovial membrane (SM) of individuals with early osteoarthritis using flow cytometry. The study revealed a significant increase in the expression of the cytokine IFN-γ in Th1 cells, while the expression of CCR3 and CCR4, primarily associated with Th2 cells, did not show a notable increase. This observation supports the notion of an altered Th1/Th2 cell balance in early knee osteoarthritis (KOA). Certain drugs, such as Sesamol (104), can regulate the balance between cellular immune responses and Th1/Th2, thereby exerting various pharmacological effects such as anti-inflammation and immune regulation. Low molecular weight polypeptide 7 (LMP7) serves as an immune proteasome subunit that influences the proliferation and specialization of T cells and modulates the balance between Th1/Th2 and Th17/Treg subsets (105). Therefore, the dysregulation of Th1/Th2 cell balance is a significant factor in the pathogenesis and progression of OA. Modulating the equilibrium of Th1/Th2 cells represents a promising therapeutic strategy for managing OA. By conducting thorough research on the functionality and interaction mechanisms of Th1 and Th2 cells, as well as investigating novel approaches to modulate this cellular equilibrium, a fresh outlook and efficient intervention for OA treatment could potentially be established. Some scholars (106) have constructed an autoantigen type II collagen peptide (CII250-270C) and the immunomodulator leflunomide (LEF) within a phosphatidylserine liposome vaccine (CII250-LEF-PSL) as a therapeutic approach for RA. This vaccine aims to promote the activation of regulatory T cells (Treg) by inducing tolerant dendritic cells (TolDC). They found that CII250-LEF-PSL effectively stimulates the differentiation of Th1 cells, modulates the Th1/Th2 balance, ameliorates synovial and cartilage damage, and consequently alleviates the symptoms of RA. Thus, the potential application of a co-delivery system involving autoantigen peptide and immunomodulator for the prevention and treatment of OA, aiming to ameliorate OA symptoms through modulation of the Th1/Th2 balance, is a promising area for further investigation.

## 5 Treg cells and OA

In the mid-1990s, a group of Th cells with regulatory functions was identified and named regulatory T cells (Treg) (107). Treg cells are actively controlled immune tolerance cells in the body’s immune system. They play a negative role in the activation and proliferation of T cells. They contribute to the maintenance of immune tolerance, prevention of autoimmune diseases, anti-graft rejection, and tumor immunity (108, 109). Treg cells can be divided into natural regulatory T cells (nTregs) and induced adaptive regulatory T cells (aTregs or iTregs). These cells can function by interacting through direct contact with cell surface molecules rather than cytokines. For instance, cytotoxic T lymphocyte-associated antigen 4 (CTLA-4) interacts with CD80/CD86, and glucocorticoid-induced tumor necrosis factor receptor (GITR) interacts with GITR ligand (GITRL), facilitating immune regulation. Tregs can also inhibit autoimmune diseases by producing inhibitory cytokines (such as TGF- β, IL-10, IL-35) (86, 110, 111). For example, by secreting IL-10, Treg cells inhibit inflammation and autoimmune reactions, thereby contributing to immunosuppression and alleviating OA symptoms. Tregs may be an ideal cell type for the targeted treatment of OA. Kim (112) studied the use of lipid nanoparticles to regulate Treg cells in an antigen-specific manner. It was found that lipid nanoparticles can regulate the expression of cytokines and reduce the infiltration of immune cells in joints, thus inhibiting apoptosis and matrix degradation in OA chondrocytes, and relieving pain. The differentiation of Treg cells inhibited the pathogenesis of OA. In an OA rat model study, Kwon et al. (113) treated the OA rats with an intraarticular injection of Gukangning. The gene expression was detected by real-time fluorescence quantitative polymerase chain reaction and the protein expression was detected by immunohistochemistry. The results demonstrated that Gukangning inhibited cartilage osteoclasts and activated joint Treg cells, thus reducing OA pain and improving cartilage destruction. Synovitis interacts with Treg cells in the early stage of OA. Keller (114) used three horse-cultured OA models to co-culture synovial cells and chondrocytes in the Transwell system to establish normal joint and osteoarthritis models. Keller found that Treg cells can increase the expression of IL-10 and IL-4 in synovial cells and chondrocytes and increase the expression of the TIMP1 gene in synovial cells and chondrocytes, indicating their potential role in protecting cartilage. Additionally, although *in vitro* results suggest enhanced Treg function upon IL-6 blockade, further studies are needed to confirm these effects *in vivo* and assess their impact on the progression of OA. An MR study using UK Biobank and GWAS data shows that CD25, especially CD4+ and CD25+T cells, have a protective effect on OA of the hip joint (115). Clinical studies (116) have shown that in patients with osteoarthritis (OA), the frequency of CD4+CD25+Foxp3hi Tregs is significantly increased in the peripheral blood compared to healthy controls. However, the secretion of IL-10, which is also produced by Treg cells, is decreased in these patients. Importantly, this reduction in IL-10 secretion is associated with decreased expression of Tim-3 on Tregs. While both Tim-3(-) and Tim-3(+) Tregs can produce IL-10, the majority of IL-10 secretion is observed in the Tim-3(+) Treg subset. In another clinical study, the researchers analyzed Treg cell infiltration in peripheral blood (PB), synovial fluid (SF), and synovial membrane (SM) of 47 patients undergoing knee arthroplasty by flow cytometry. Knee joint pain and joint function were evaluated and correlated with the proportion of Treg cells from different sources (peripheral blood, synovial fluid, synovium). It was found that the proportion of Treg cells in the joint samples was significantly higher than that in the peripheral blood samples. A significant correlation between infiltrating Treg cells and OA-related symptoms was also observed (117). The above studies confirmed that an imbalance of Treg cellular immunity occurs in patients with OA. Treg cells participate in the pathogenesis of OA by modulating inflammatory responses that contribute to joint degeneration. Consequently, Treg cells impact OA in significant ways.

## 6 The imbalance of Th17/Treg cells is an important mechanism of OA

**Figure 3:**
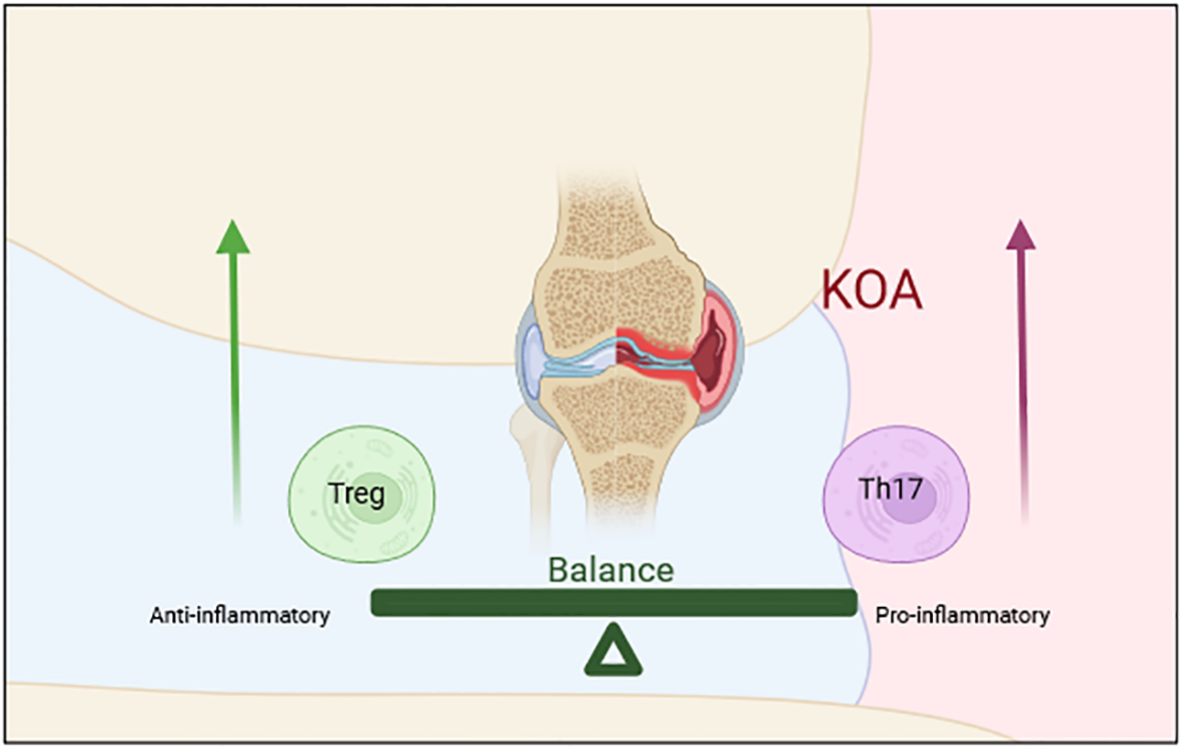
The imbalance of Th17/Treg cells is an important mechanism of OA. Th17 cells promote inflammatory responses and represent the pro-inflammatory subsets, while Treg cells inhibit inflammatory responses and antagonize the function of Th17 cells. The imbalance of Th17/Treg cells is involved in the pathophysiological processes of OA.

Th17 cells and Treg cells develop from the same immature CD4+T lymphocytes. A complex relationship exists between them. Th17 cells promote inflammatory responses and represent the pro-inflammatory subsets, while Treg cells inhibit inflammatory responses and antagonize the function of Th17 cells (118). They also inhibit each other in differentiation. Studies have shown that Treg cells can inhibit the differentiation of Th17 cells by up-regulating the specific transcription factor Foxp3 or down-regulating the expression of IL-23 and IL-17. Similarly, inhibition of Th17 cells can promote the development of Treg cells (119, 120). The differentiation of CD4+T cells is a highly complex process. Activation of the cellular microenvironment and signal pathway directly determines the differentiation of CD4+T cells into the different subsets, which in turn, affects the balance of Th17/Treg cells. Th17 cells mainly secrete IL-17, which is one of the early initiating factors of joint inflammation, with a strong pro-inflammatory effect. Treg cells mainly secrete TGF-β and IL-10 to inhibit the function of self-reactive lymphocytes, thus exerting an immunosuppressive role (121–123). The balance of Th17/Treg cells maintains the balance of human immunity, and it is strictly regulated under healthy conditions (124). In the early stage of OA, the homeostasis of the joint becomes gradually out of balance under the stimulation of persistent inflammatory factors. An imbalance of Th17/Treg cells is found in patients with OA, and the proportion is closely related to OA progression (118, 125). Mansoori et al. (126) confirmed that in the ovariectomized mouse model, macrophages and CD4+T cells not only induce periodontal disease in mice but also secrete pro-inflammatory cytokines to induce NLRP3 inflammatory bodies in osteoblasts and increasing the Th17/Treg ratio, thus aggravating the formation of osteoclasts and aggravating the destruction of subchondral bone. Other researchers have confirmed that osteocytes can produce immunomodulatory cytokines through NLRP3 inflammatory bodies, change the ratio of Th17/Treg cells and osteoclast production, and thereby, aggravate the immune response, leading to bone destruction and joint degeneration (122). Ponchel et al. (71) analyzed the blood of 114 patients with OA and 121 healthy controls. In this study, Treg cells were significantly lower in patients with OA than that in the controls. The results also demonstrated that CD+T cells differentiated into Th17 cells in the synovium of those with OA. In another clinical study comparing patients with OA treated with saffron and a blank control group, the level of Th17 cells in the peripheral blood of those with OA decreased significantly, but the level of Treg cells increased (127). These studies confirmed that under normal conditions, the effects of Treg cells and Th17 cells are in a relatively balanced state, while an imbalance in the proportion of Th17/Treg cells is observed in patients with OA. IL-6 plays an important role in determining the direction of T cell differentiation. Its absence promotes the differentiation of immature CD4+T cells into Treg cells, while its existence promotes the differentiation of Th17 cells (128, 129). In sum, Th17/Treg cells have a key role in the development of OA, and the imbalance of Th17/Treg cells is involved in the pathophysiological processes of OA (**Figure 3**).

## 7 Regulating the imbalance of Th17/Treg cells is the key target for the treatment of OA

**Figure 4:**
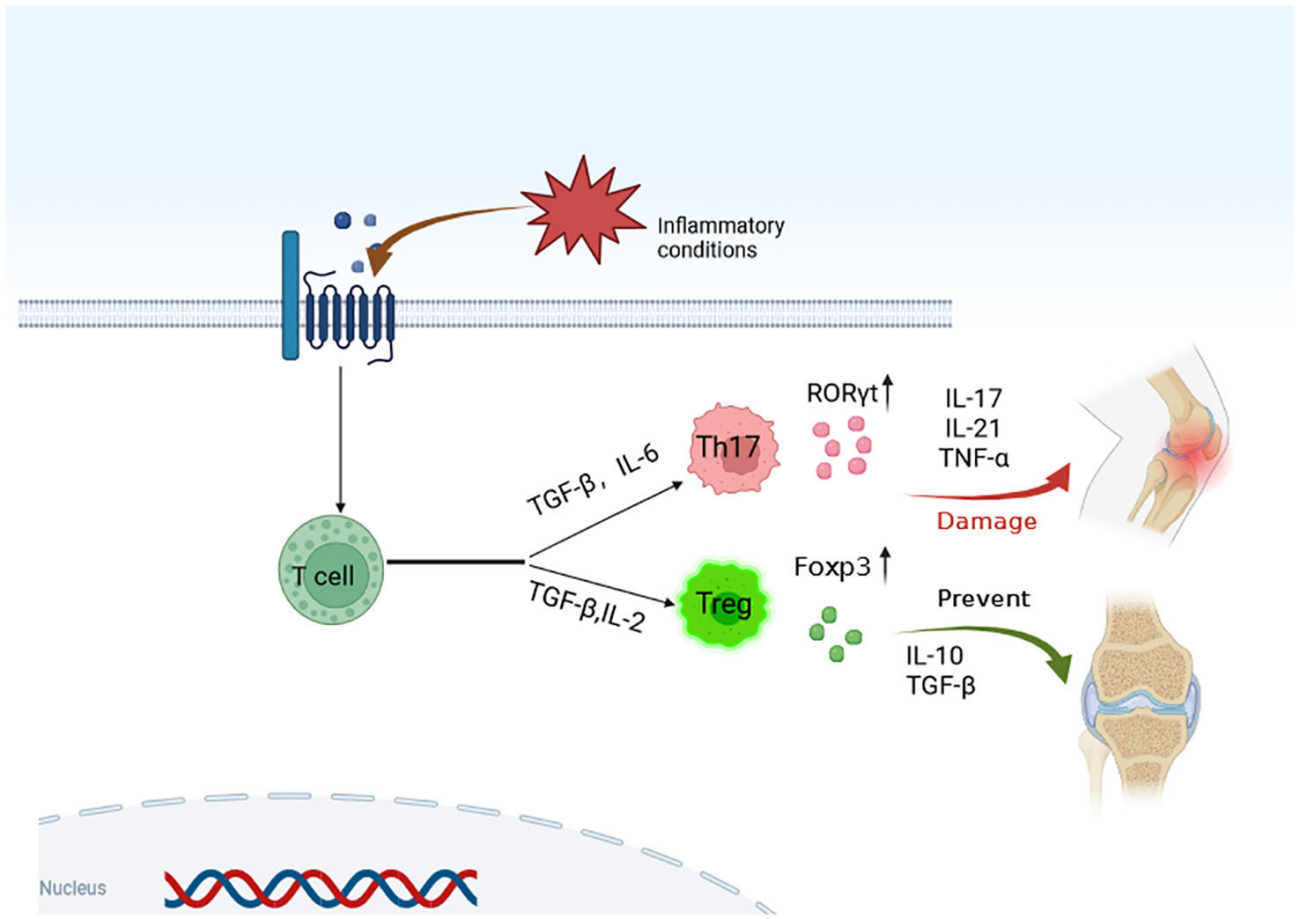
Modulating Th17/Treg cell imbalance is a key target for the treatment of OA. Through targeted regulation of cytokines and transcription regulators, the differentiation of T cells is regulated, and the activation and function of Th17/Treg cells are affected, so as to achieve the purpose of OA treatment.

Th17 cells are pro-inflammatory CD4+ effector T cells, while Treg cells are specialized T cell with immunosuppressive and anti-inflammatory effects (130, 131). In the pathological process of OA, the balance between Th17 cells and Treg cells is disrupted, leading to inflammatory reactions and the destruction of the articular cartilage. Considering this, the Th17/Treg balance may be a potential target for new OA treatment (129, 132, 133). For example, some studies have confirmed that the proportion of Th17 cells in the synovial fluid and peripheral blood of patients with OA increases, while the proportion of Treg cells decreases, indicating that Th17/Treg imbalance plays an important role in the pathogenesis of OA. By regulating the balance of Th17/Treg cells, inflammatory responses are reduced, thus potentially relieving pain and improving joint function in models of OA, as suggested by studies (125, 134). At present, many treatments addresses the imbalance of Th17/Treg cells. Some non-steroidal anti-inflammatory drugs (NSAIDs) can inhibit the synthesis of prostaglandins, and thus, reduce the inflammatory response. Using an induction of spinal arthritis (SPA) mice model, Min et al. investigated vitronectin-derived bioactive polypeptide NPP-16 combined with celecoxib as treatment and found that VNP-16 combined with celecoxib prevented the progression of SPA by regulating the balance of Th17/Treg cells and inhibiting the expression of pro-inflammatory cytokines (135). Another study found that NSAIDs, such as ibuprofen and indomethacin, can alleviate pain and inflammation by modulating Th17/Treg imbalance in OA models (136). Some cytokines, such as IL-1, IL-6, and TNF- α, modulate the inflammatory responses in OA. Targeted drugs for these cytokines can regulate the balance of Th/Treg cells and reduce joint inflammatory responses in OA models, which may lead to pain relief and improvement in joint function (131, 137, 138). Additionally, some immunomodulatory drugs can regulate Th17/Treg balance and relieve the symptoms of OA. For example, statins can inhibit the differentiation of Th17 cells and promote the production of Treg cells, thus regulating Th17/Treg balance (139, 140). The differentiation of Treg cells requires the inactivation of mammalian rapamycin target (mTOR) and the activation of AMP-activated protein kinase (AMPK). Peroxisome proliferator-activated receptorγ (PPARγ) is a nuclear receptor that regulates Th17/Treg balance. Therefore, Th17 is transferred to Treg cells by activating AMPK and PPARγ, thus regulating Th17/Treg balance (129, 141). Tawfeek (142) prepared collagen-coated PCL nanofibers and characterized them by scanning electron microscope to study the effect of nanofiber scaffolds on Th17/Treg immunomodulatory properties of bone marrow mesenchymal stem cells in osteoarthritis and its mechanism. The nanofiber scaffolds enhanced the immunomodulatory effect of the bone marrow mesenchymal stem cells in osteoarthritis by increasing the expression of intercellular adhesion molecules. The treatment of Th17/Treg cell imbalance may become a key target of OA (**Figure 4**). It can relieve pain and improve joint function by regulating Th17/Treg balance and reducing inflammatory response in OA patients. Therefore, regulating the imbalance of Th17/Treg cells is the key target for the treatment of OA. Intervention at different levels that target different cytokines, transcriptional regulatory factors, and apparent modifications can affect the activation and function of Th17/Treg cells. By regulating the inflammatory environment, OA symptoms are improved. These targets should be considered as potential new targets for the treatment of OA.

## 8 Discussion and prospects

Th and Treg cells are important subsets of T lymphocytes. They are important in the pathogenesis of OA. Th cells mainly include Th1, Th2, Th9, Th17, and Th22 subsets, which regulate immune responses by secreting different cytokines. Th1 cells are mainly involved in cellular immune responses, Th2 cells are mainly involved in humoral immune responses, and Th17 cells are mainly involved in inflammatory responses. Treg cells are important immunomodulatory cells, which can inhibit immune responses and maintain immune homeostasis. In OA, there is also a complex interaction between Th/Treg cells and other immune cells. In the presence of an imbalance inflammatory reactions and joint injury arise. For example, Th1 and Th17 cells can promote the activation and differentiation of macrophages, which in turn, promote inflammatory responses and joint injury. Conversely, Treg cells can inhibit the activation and differentiation of macrophages, thus inhibiting inflammatory reactions and joint injury. Th/Treg cells can also interact with other immune cells, such as B lymphocytes and natural killer cells, to regulate immune and inflammatory responses. An increased level of inflammatory factors, such as IL-1, IL-6, and TNF- α in the synovial fluid of patients with OA can activate and increase Th1 and Th17 cell functions, resulting in inflammatory responses and joint injury. while Treg cells inhibit inflammatory responses and joint injury by secreting anti-inflammatory factors, such as IL-10. The activation and function of Th/Treg cells can be affected by regulating the joint inflammatory environment. By optimizing Th/Treg cell functions, the symptoms and pathological changes of OA may be improved. In conclusion, the dysregulation of Th1/Th2 and Th17/Treg ratios is a crucial factor in the development of OA. By delving deeper into the mechanisms of these cell populations and their interactions, we can uncover fresh insights and potential targets for the early detection and treatment of OA (143).

As Th/Treg cells play a key role in the pathogenesis of OA, targeted therapy to optimize Th1/Th2 and Th17/Treg cell balance may be a potentially new strategy for OA therapy. At present, drugs, such as anti-tumor necrosis factor (TNF) inhibitors, are being used to treat patients with OA. These drugs can inhibit inflammation and relieve symptoms, such as pain. Further studies to determine if common treatment of OA, such as glucosamine and chondroitin sulfate, can promote the repair and regeneration of chondrocytes by regulating the imbalance of Th/Treg is warranted. Other drugs that can regulate the activation and function of Th/Treg cells by inhibiting or promoting the expression level of specific molecular markers should also be considered. In the future, research is needed to explore the application prospect of Th/Treg balance therapy in the treatment of OA.
